# Thalamocortical Communication in the Awake Mouse Visual System Involves Phase Synchronization and Rhythmic Spike Synchrony at High Gamma Frequencies

**DOI:** 10.3389/fnins.2018.00837

**Published:** 2018-11-22

**Authors:** Samuel S. McAfee, Yu Liu, Mukesh Dhamala, Detlef H. Heck

**Affiliations:** ^1^Department of Anatomy and Neurobiology, University of Tennessee Health Science Center, Memphis, TN, United States; ^2^Department of Diagnostic Imaging, St. Jude Children’s Research Hospital, Memphis, TN, United States; ^3^Department of Physics and Astronomy, Neuroscience Institute, Georgia State University, Atlanta, GA, United States

**Keywords:** gamma oscillations, high gamma, mouse, neuronal synchronization, visual system

## Abstract

In the neocortex, communication between neurons is heavily influenced by the activity of the surrounding network, with communication efficacy increasing when population patterns are oscillatory and coherent. Less is known about whether coherent oscillations are essential for conveyance of thalamic input to the neocortex in awake animals. Here we investigated whether visual-evoked oscillations and spikes in the primary visual cortex (V1) were aligned with those in the visual thalamus (dLGN). Using simultaneous recordings of visual-evoked activity in V1 and dLGN we demonstrate that thalamocortical communication involves synchronized local field potential oscillations in the high gamma range (50–90 Hz) which correspond uniquely to precise dLGN-V1 spike synchrony. These results provide evidence of a role for high gamma oscillations in mediating thalamocortical communication in the visual pathway of mice, analogous to beta oscillations in primates.

## Introduction

Sensory processing requires selective and effective communication between neuronal groups, and this is influenced by the temporal alignment of regional oscillations (Fries et al., 2007; Womelsdorf et al., 2007; Bastos et al., 2015; Fries, 2015). In the primary visual cortex (V1), local field potential (LFP) oscillations are stimulus-dependent, meaning specific image properties evoke oscillations with certain frequency and power characteristics (Henrie and Shapley, 2005; Ray and Maunsell, 2010; Roberts et al., 2013). Notably, stimuli can evoke oscillations in distinct frequency bands simultaneously, which may represent different forms of information coding, and/or parallel channels for neuronal communication (Colgin et al., 2009; Bastos et al., 2014; Richter et al., 2017). Much attention has been given to these oscillations and the role they play in cortico-cortical communication for visual processing (Usher and Donnelly, 1998; Engel et al., 2001; Veit et al., 2017a), but less is known about their possible role in the relay of visual information from the dorsolateral geniculate nucleus of the thalamus (dLGN) to V1 in awake animals.

The current body of evidence suggests there are species-specific differences in network oscillation mechanisms and frequencies (Castelo-Branco et al., 1998; Bastos et al., 2014). In primates, thalamocortical communication is supported by synchronized oscillations in the beta frequency range (15–25 Hz), with gamma oscillations first emerging in the cortex (Bastos et al., 2014). By contrast, recent studies in mice show that a prominent narrowband oscillation (2–5 Hz bandwidth) in the high gamma range (55–70 Hz) underlies visual thalamocortical communication for luminance information (Saleem et al., 2017; Veit et al., 2017a). However, the introduction of visual contrast leads to suppression of the narrowband oscillation in V1 and the emergence of two distinct broadband oscillations (>20 Hz bandwidth) in the low gamma (25–35 Hz) and high gamma (50–90 Hz) ranges. It remains unclear what role these distinct gamma oscillations play in visual processing. We hypothesized that one oscillation may be related to thalamocortical communication, and therefore rhythmic neuronal activity in this band would occur synchronously in dLGN and V1 during the presentation of high contrast visual stimuli.

We addressed this by performing dual dLGN-V1 recordings of LFPs and spikes in awake, head-fixed mice, and asked whether high contrast images induce synchronized band-limited gamma oscillations in mouse dLGN and V1. We further investigated whether these V1 oscillations were linked to precisely coincident spikes in V1 and dLGN; evidence of oscillatory neuronal communication between the two structures. High-contrast visual stimulation led to synchronized dLGN and V1 high gamma oscillations that were temporally aligned with coincident spikes. These findings suggest that high gamma in mouse V1 constitutes a channel for thalamocortical communication, and this gamma rhythm may be frequency- and bandwidth-modulated by stimulus properties such as luminance and contrast.

## Materials and Methods

### Animals

Experiments were performed in six male and female (2 female, 4 male) C57BL/6J mice (>8 weeks old, 18–28 g body weight). Mice were housed in a breeding colony with 12-h light/dark cycles with ad libitum access to food and water. All mice were housed in the dark for 12 h prior to recording. All experimental procedures adhered to guidelines approved by the University of Tennessee Health Science Center Animal Care and Use Committee. Principles of laboratory animal care (NIH publication No. 86-23, rev. 1996) were followed.

### Preparation for Awake, Multisite Recordings

Mice were induced for surgery with 3% isoflurane (Baxter Pharmaceutical Products, Deerfield, IL, United States) in oxygen in an incubation chamber, and transferred to a stereotaxic head mount. Anesthesia was maintained with 1–2.5% isoflurane in oxygen through a nose cone. Isoflurane concentration was controlled with a vaporizer (Highland Medical Equipment, Temecula, CA, United States). The depth of anesthesia was adjusted until the mice failed to show a reflex withdrawal of the hind paw to a strong pinch. Surgical techniques were described in detail elsewhere (Bryant et al., 2009). In brief, a small craniotomy (2 mm × 1 mm) was made above the primary visual cortex (Bregma -3.38 mm, lateral 2.25 mm) and dorsal lateral geniculate nucleus (Bregma -2.18 mm, lateral 2.0 mm). The exposed but intact dura was covered with Triple Antibiotic (Walgreens, United States) to maintain moisture and reduce the risk of infection. A cylindrical plastic chamber (0.45 cm diameter and 4 mm height) was placed over the skull opening and filled with Triple Antibiotic and Kwik-sil epoxy (World Precision Instruments, Sarasota, FL, United States). Three small machine screws (1/8^′^ dome head, 0.8 mm diameter, 2 mm long, Small Parts, Inc., Miami Lakes, FL, United States) were secured in the skull bone, and a metal head post was mounted anterior to Bregma. The chamber, head post and skull screws were secured in place with super glue and dental acrylic. Mice were injected subcutaneously with 5 mg kg^-1^ analgesic Carprofen (Zoetis Inc.; Kalamazoo, MI, United States) to alleviate pain and 0.25 ml of lactated ringer solution as a fluid supplement twice within the first 24 h of the surgery. Mice were allowed a 4-day recovery from surgical preparation before subsequent procedures.

### Electrophysiological Recordings

Mice were habituated to head fixation above a freely rotating treadmill for one 30-min session before recording sessions started. The experimental setup, head-holding device and recording procedures have been described in detail previously (Bryant et al., 2009). Head fixation was accomplished by tightening a mechanical screw attached to a fixed bar through a threaded hole in the metal head post. Mice were allowed to move and walk freely on the treadmill at all times. The plastic chamber was then cleaned and filled with sterile saline solution.

LFP recordings were obtained using five hand-made glass-insulated tungsten/platinum electrodes (impedance 1.0–5.0 MΩ) guided in the ventral-dorsal direction by a computer-controlled microdrive (MiniMatrix, Thomas Recording, Germany). Stainless steel microdrive guiding tubes served as reference electrodes, and were electrically connected to the brain tissue via the electrolyte solution in the recording chamber. Guiding tubes were positioned at the surface of the brain above the targeted structures, and the electrodes were slowly advanced through the dura to their approximate target depths. Locations of electrode tips in layer 4 of V1 and dLGN were preliminarily determined based on established patterns of visually evoked neuronal activity (Niell and Stryker, 2008). Electrode movements were controlled with micrometer resolution and digitally monitored (TRec EMM Client, Thomas Recording, Germany). LFP and spike signals were separated by band pass filtering at 0.1–200 Hz and at 200 Hz to 8 kHz, respectively, using a hardware filter amplifier (FA32; Multi Channel Systems). Filtered and amplified voltage signals were digitized and stored on a computer hard disk (16 bit A/D converter; sampling rate, >20 kHz for action potentials, >2 kHz for LFPs) using a CED power1401 and Spike2 software (both Cambridge Electronic Design).

Upon completion of each recording session, electrolytic lesions were created in the tissue at the electrode tips to confirm recording sites in slice. All mice were euthanized within 8 h of recording and transcardially perfused with phosphate-buffered saline and 4% paraformaldehyde prior to brain tissue collection.

### Presentation of Visual Stimuli

Visual stimuli were designed and presented using customized Psychtoolbox and Matlab scripts (Brainard, 1997; Pelli, 1997). Checkerboard images (100% contrast, 0.05 cpd, 100 ms duration) were presented on a 22^′′^ computer monitor centered 30° lateral to the animal’s midline and 25 cm away. Stimuli were presented at randomly generated intervals ranging from 2.5 to 4 s. During inter-stimulus intervals the monitor remained gray, with the same average luminance as the checkerboard. Precise timing of visual stimuli was co-registered to the LFP by simultaneously recording the voltage from a photodiode placed on the corner of the screen. LFP recordings were taken on the contralateral side to the visual field in which stimuli were presented.

### Analysis of LFP Amplitude and Phase

Recorded data were visually inspected and periods that were free of movement artifact or treadmill rotation from locomotion were selected for further analysis. 60 Hz contamination of the signal by ambient electrical noise was removed using a hum-removal algorithm in the Spike2 software (HumRemoveExpress.s2s). LFP data and stimulus times were then exported to Matlab for further analysis.

For LFP analyses, frequency components of each LFP signal were extracted using a causal FIR band pass filters. Filters of bandwidth 0.5–5 Hz were used in 0.5 Hz steps, with filter order equal to the number of samples in 5 cycles for each center frequency. An amplitude time series for each frequency was estimated by applying the Hilbert transform to each band pass filtered signal and taking the absolute value of the analytic signal. Changes in amplitude were calculated as a percent difference from the mean values occurring within 1 s prior to the stimuli. Instantaneous phase values were determined at each time point as the angular value of the analytic signal. Pairwise-phase consistency (PPC) (Vinck et al., 2010) values were calculated on phase relationships across trials for each frequency and latency from the stimulus in 1ms time steps.

### Time Resolved LGN-V1 Spike Synchrony

To assess the connection between oscillatory synchrony and neuronal communication between V1 and dLGN neurons, we focused on the occurrence of synchronous firing of single units between the regions. Single units were detected using the offline spike sorting algorithm in Spike2, and spike times were exported to Matlab for further analysis.

A major aim of this study was to determine whether the occurrence of synchronous spikes corresponded to specific frequencies of oscillations in the LFP. To do this effectively, statistical increases in coincidence due to increases in rate must be corrected for, as the rate of transmission has an important but separate contribution to the effectiveness of neuronal communication. To accomplish this, we created a continuous time series reflecting spike synchrony by sliding a time window across the recording period and measuring the coincidence of V1 and dLGN spikes (e.g., the product of their counts) within the window. We shifted the time of dLGN spikes by 5 ms to account for the known conduction delay (MacLean et al., 2006), and measured coincidences within a conservative 4.5 ms time window corresponding to the interval within which synchronous output from dLGN evokes maximal V1 responses (Wang et al., 2010). The mean result derived from 200 surrogate data sets generated by random spike jittering was then subtracted to correct for synchrony occurring due to increases in spike rate alone (±2–10 ms jitter range, 0.5 ms resolution, uniform probability). The jittering method was found to be preferable to using a trial shift or shuffle predictor for this correction, as it disrupted only the precise timing of spikes while preserving the approximate latency and specific spike count in each response (Torre et al., 2016). The time series was then smoothed with a 4.5 ms window. This process revealed rhythmicity in the synchronous occurrence of spikes, which could then be compared to oscillations in the LFP.

### Correlation of Spike Synchrony and Gamma Activity

Lastly, we investigated whether synchronously occurring spikes in dLGN and V1 were consistently linked to the occurrence of V1 oscillations at different frequencies. To do this, we performed a cross correlation analysis between the spike synchrony time series and different frequencies of oscillations in the V1 LFP. Sections corresponding to gray screen and checkerboard viewing were separated before further analysis to evaluate possible differences between the two states.

### Experimental Design and Statistical Analysis

Six mice successfully underwent multisite electrophysiological recording in the current study. Successful recording was defined as having one or more units in each location that responded positively to contrast increase, having at least 250 artifact-free stimulus cycles executed over a 15 min recording session, and having histological confirmation of recording in dLGN and layer 4 of V1. Twelve mice underwent at least some portion of the aforementioned surgical and experimental procedures but failed to meet these criteria.

All statistical analyses were performed in Matlab. Statistical assessment of correlations between LFP oscillations and spikes was performed using non-parametric Monte Carlo approaches with surrogate data. For assessment of the correlation between PPC and V1 oscillation amplitude, surrogate values were generated for each evaluated frequency by randomly shifting PPC results across time in a circular manner with respect to the mean LFP amplitude of the same frequency. Two thousand surrogate values were generated for each frequency for each animal. The surrogate coefficients were then ranked, and the 95th and 5th percentile coefficients for each frequency were used as a statistical threshold. Averaging of correlation values for graphical representation was performed following a Fischer transformation of the coefficients, and then the inverse on the mean. *R*^2^ values for the peak V1 high gamma frequencies were created by taking the square of the raw coefficient, and averaged across animals.

For analysis of the relationship between LGN-V1 spike synchrony and the V1 LFP, we developed a Monte Carlo hypothesis test for the precision of spike times and their temporal relationship to V1 oscillations in the high gamma range (50–90 Hz). For this approach, real correlations were tested against a ranked distribution of surrogate correlation values between the V1 LFP and jittered spike synchrony waveforms. To conservatively test for a relationship between thalamocortical spike synchrony and V1 gamma without bias caused by the phase locking of V1 spikes to V1 gamma, only dLGN spike times were jittered in the generation of surrogates. Correlation values above the 95th percentile or below the 5th percentile are considered significant.

## Results

### Visual Stimulus-Driven Gamma Oscillations in dLGN and Layer 4 of V1

**FIGURE 1:**
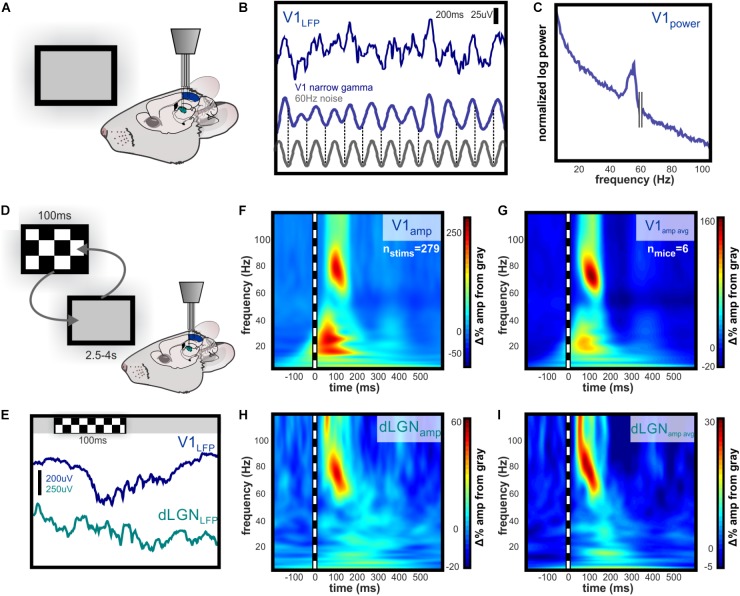
Evoked narrowband and broadband high gamma oscillations dLGN and V1. Independently guided electrodes were used to record LFP and spikes from L4 of V1 and dLGN during visual stimulation. **(A)** Illustration of experimental setup during baseline recording of activity evoked by viewing of the gray computer screen. **(B)** Example of 200 ms of raw narrowband gamma in L4 of V1 evoked by luminance of a gray computer screen. The frequency is between 50 and 60 Hz, but ambient 60 Hz electrical noise is not linked to this oscillation, as illustrated by the phase shifts near the beginning and end of trace. **(C)** Spectral power of narrowband gamma oscillation in V1. Peak in spectral power is apparent below 60 Hz. **(D)** Illustration of experimental setup during randomized presentation of high contrast checkerboard image. **(E)** Example raw LFP in V1 and dLGN during single checkerboard presentation. Contrast-evoked oscillations are higher in amplitude and wider in bandwidth than narrowband gamma in V1, and are also visible in dLGN. **(F)** Example time-frequency analysis of contrast-evoked LFP oscillations in V1, normalized to amplitude during gray screen viewing. 100 ms checkerboard image evokes high amplitude oscillations in distinct low and high gamma frequency ranges. **(G)** Group mean change in oscillation amplitude over time relative to LFP during gray screen viewing. Peak amplitude change occurs in the 50–90 Hz range 100–125 ms after stimulus onset. **(H,I)** Same as **(F,G)** for LFP in dLGN. Low gamma amplitude is not contrast-modulated in dLGN, and high gamma amplitude reaches near-maximal value before V1.

Replicating results reported in other studies, we observed high amplitude gamma oscillations in layer 4 of V1 in response to a high-contrast checkerboard image (Niell and Stryker, 2008; Saleem et al., 2017; Veit et al., 2017a) as well as “narrowband” gamma oscillations in response to a gray screen (Saleem et al., 2017) (Figures 1A–E). To identify oscillatory changes in V1 and dLGN associated with contrast, we performed time-frequency analysis of LFPs, normalized to oscillation amplitudes during gray screen presentation (Figures 1F–I). V1 oscillation amplitude increased in the high beta/low gamma (20–35 Hz) and high gamma ranges (50–90 Hz), the latter of which was higher in frequency and broader in bandwidth than previously reported narrowband gamma (Saleem et al., 2017; Veit et al., 2017b). The dLGN LFP showed no such increase in beta or low gamma but did show increased high gamma amplitude, with frequencies matching those in V1. On average, high gamma amplitude increased more rapidly, and reached a near-maximal value in dLGN before V1 (Figures 1G,I).

### Phase Synchronization and Amplitude of LFP Oscillations

**FIGURE 2:**
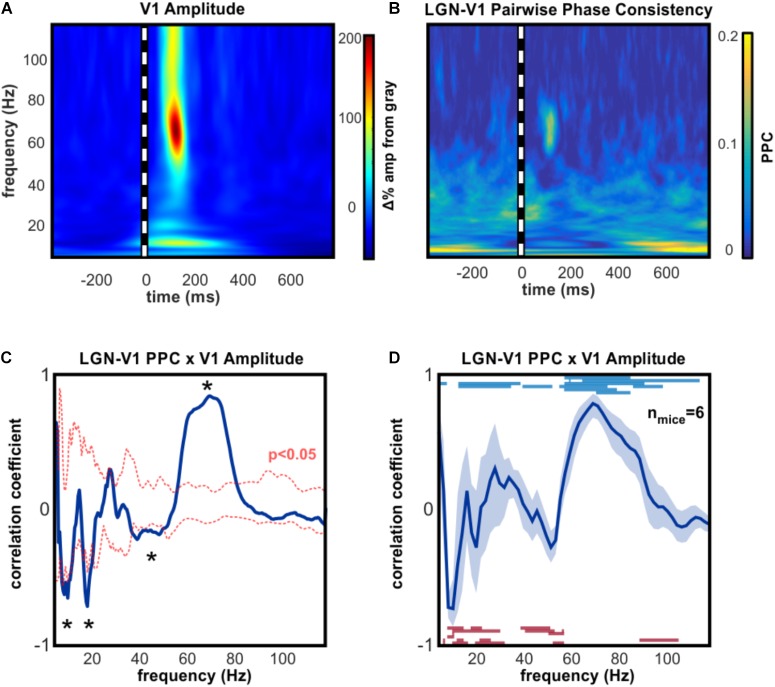
Amplitudeof high gamma V1 oscillations is linked to simultaneous thalamic high gamma. **(A)** Example time-frequency analysis of V1 LFP, aligned on the presentation of 100 ms full-contrast checkerboard image. **(B)** Example time-resolved pairwise phase consistency analysis of oscillation phase synchronization between dLGN and V1. PPC is increased in the high gamma frequency range following checkerboard stimulus. **(C)** Example correlation analysis of **(A,B)** to identify V1 oscillations whose amplitude is dependent on synchronous thalamic oscillations across stimulus conditions. Asterisks indicate frequencies of significant correlation (*p* < 0.05). High gamma in V1 is strongly correlated with synchronous high gamma oscillations in dLGN, and strongly anti-correlated with synchrony in the alpha, beta, and low gamma ranges. **(D)** Group mean results (±SEM) for correlation analysis shown in **(C)**. Blue lines show frequencies of significant positive PPC-amplitude correlations in individual mice. Red lines show frequencies of significant inverse PPC-amplitude correlation. All animals had significant correlations within the 50–90 Hz high gamma frequency range and significant inverse correlations in the alpha frequency range. At the peak high gamma frequency in V1, dLGN-V1 phase synchronization accounted for an average of 66.9% of the amplitude modulation, across animals.

Gamma oscillations in the cortex are most commonly generated and maintained by local inhibitory feedback (Fries et al., 2007; Whittington et al., 2011; Buzsaki and Wang, 2012), however narrowband high gamma activity in V1 can be driven by excitatory input from dLGN, even when cortex is silenced (Saleem et al., 2017). To explore the hypothesis that broadband high gamma oscillations occurring in V1 were driven by dLGN, we investigated which V1 oscillations increased in amplitude while synchronized with dLGN oscillations. A correlation analysis was performed on the average time course of phase synchronization (PPC) and the amplitude of oscillations in V1 (Figures 2A,B), an approach that was developed for a comparable investigation in primates (Bastos et al., 2014). This analysis revealed that V1 high gamma amplitude co-varied significantly with the degree of phase synchronization with dLGN (Figures 2C,D). Phase synchrony accounted for an average of 66.9% of the amplitude variance in V1 high gamma across animals. A consistent inverse pattern was found in the alpha range, such that alpha oscillations in V1 became uncoupled from those in dLGN as contrast drove alpha amplitude increases in V1.

### dLGN-V1 Spike Synchrony Is Frequency-Matched to V1 High Gamma Oscillations

We developed a method of time-resolved spike synchrony analysis to determine whether synchronous spikes were rhythmic and in concurrence with band-limited LFP oscillations. This analysis creates a continuous function that highlights synchronized spike events within a physiologically meaningful time window, with correction for spike rate.

**FIGURE 3:**
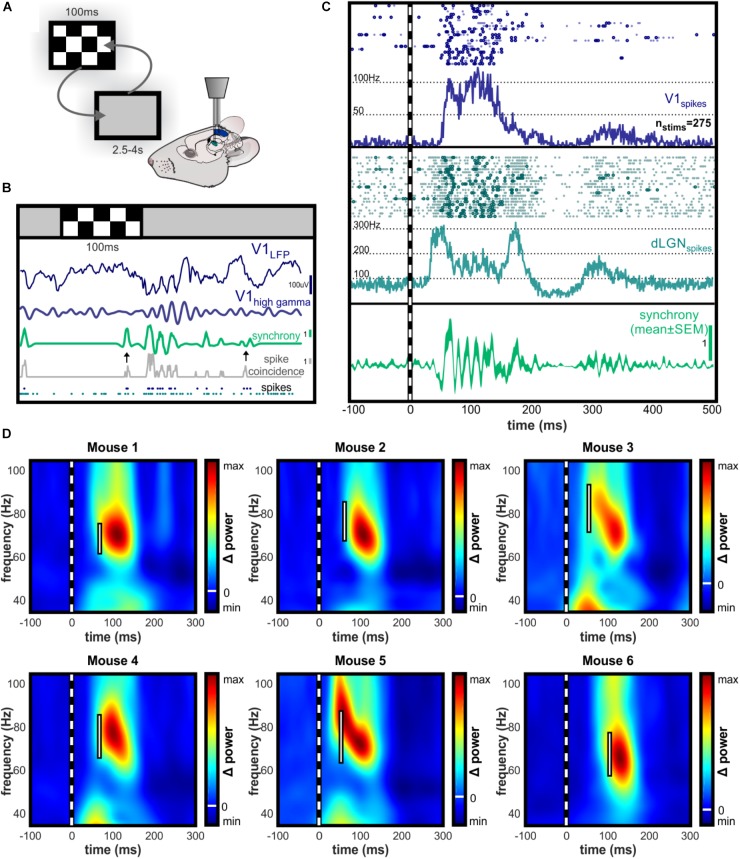
Time-resolved spike synchrony is rhythmic in the high gamma range. **(A)** Illustration of experimental setup during randomized presentation of high contrast checkerboard image. **(B)** Single trial example of dLGN-V1 spike synchrony and V1 LFP following checkerboard stimulus. Synchronous bursts of spikes are observed following presentation of the checkerboard image, which align with the gamma oscillation observed in V1. Gray trace represents the number of dLGN-V1 spike coincidences within a sliding 4.5 ms window. Green trace represents time-resolved spike synchrony, which is taken from the coincidence rate and adjusted for spike rate using spike-time jittering (see Section “Materials and Methods” for details). Scale bar shows height of 1 coincidence above mean probability for the concurrent spike rate within the jittering time window (±10 ms). Note the two black arrows indicating moments of equal spike coincidence, but high (left) versus low (right) spike synchrony values after the jitter correction is applied. This highlights the sensitivity of the method to the precise alignment of spikes rather than increases in spike rate. **(C)** Example raster plot for V1 and dLGN spikes occurring in 20 consecutive trials, and peri-stimulus histogram over all trials. Dark circles in raster plot highlight spikes occurring synchronously above the chance probability for the concurrent spike rate. After initial dLGN burst and V1 response, dLGN spikes appear to be modulated at an approximate 68 Hz rhythm. Bottom panel shows synchrony function averaged over trials to reveal rhythmicity of synchronous spikes locked to the stimulus onset. **(D)** Peak amplitude of high gamma oscillations in V1 LFP occurs following the peak of rhythmic spike synchronization in the same frequency range. Individual examples of contrast-evoked gamma oscillations with latency and bandwidth of peak spike synchrony superimposed. White bar spans the bandwidth of 95% maximal amplitude of rhythmic spike synchrony. All color axes are normalized to maximum amplitude increase and decrease with white line indicating zero amplitude change.

The raw function reveals rhythmic synchrony in visual-evoked spikes (Figures 3A,B) and the event-triggered averaging reveals this as a consistent phenomenon (Figure 3C). Sudden changes in a visual scene are known to drive rapid burst-firing of neurons in the dLGN (Grubb and Thompson, 2005), and we observed this characteristic bursting in response to the checkerboard image (Figure 3C). Time-frequency analysis of spike synchrony revealed peak rhythmicity in the high gamma range, overlapping in frequency with evoked V1 high gamma oscillations in all animals despite individual variation in oscillation frequency (Figure 3D).

### dLGN-V1 Spike Synchrony and V1 Oscillations

**FIGURE 4:**
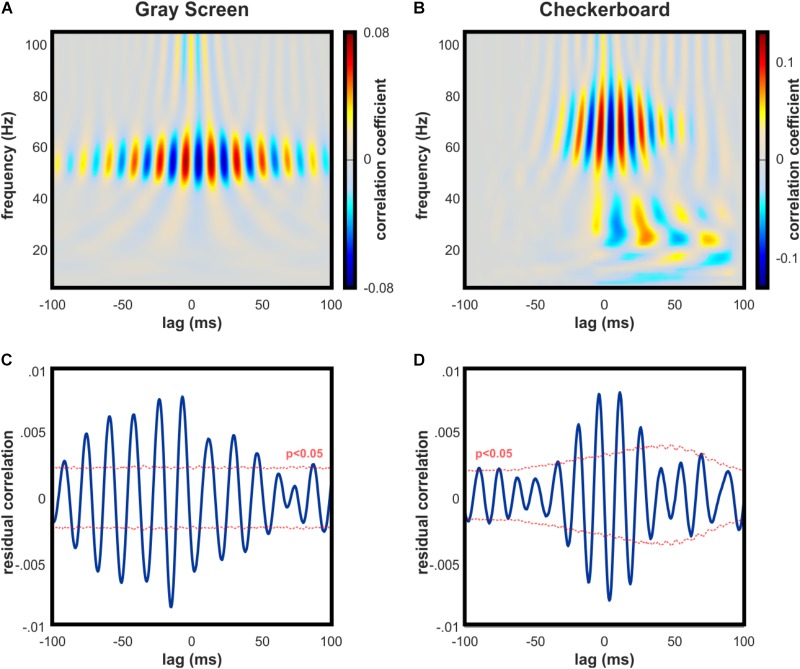
Synchronization of spike activity in dLGN and V1 is strongly linked to both narrow and broadband high gamma in V1. **(A)** Example cross-correlation of dLGN-V1 spike synchrony and V1 LFP oscillations across frequency bands during gray screen viewing. Peak correlation occurs in the narrowband gamma frequency range. **(B)** Same as **(A)** for checkerboard viewing. Peak correlation exists across a broader frequency band, consistent with contrast-evoked broadband high gamma in V1. **(C)** Example statistical test for correlation between spike synchrony and bandpass-filtered high gamma in the V1 LFP (50–90 Hz) during gray screen viewing. Tested individually, significant peaks and troughs were detected in 5 of 6 animals. Thousand surrogates were generated by jittering dLGN spikes, and median surrogate values were subtracted for easier visual interpretation. **(D)** Same as C for checkerboard viewing. Tested individually, significant peaks and troughs were detected in all animals.

To test whether V1 oscillations and spikes were temporally aligned with dLGN activity, we performed a correlation analysis between frequency components of the V1 LFP and time-resolved values of dLGN-V1 spike coincidence. This analysis was performed separately for periods where the animals were observing the gray or checkerboard images. Although average spike rates in V1 were lower during gray screen presentations, spontaneous increases in spike activity included rhythmically synchronized spikes that were uniquely correlated with narrowband gamma in V1 (Figure 4A).

Coincident spikes occurring in response to the checkerboard image were highly correlated with V1 gamma oscillations across a broader frequency band, consistent with broadband high gamma (Figure 4B). Correlation values were statistically significant in the high gamma range during visual stimulation with the checkerboard in 6 of 6 mice (Figure 4D), and during gray screen viewing in 5 of 6 mice (Figure 4C), based on the results of our Monte Carlo hypothesis test.

## Discussion

We used simultaneous recordings of LFP and spike activity in layer 4 of V1 and dLGN of six mice to identify shared patterns of rhythmic activity associated with thalamocortical communication during visual processing. Whereas previous reports advocated for a functional distinction between narrowband and broadband high gamma in V1, and were inconclusive on the role of the latter, our findings support the hypothesis that both narrowband and broadband high gamma oscillations are associated with the effective thalamocortical communication of visual information. Across stimulus conditions, the amplitude of high gamma in V1 was strongly linked to the degree of phase synchronization with high gamma in dLGN, and the occurrence of synchronous spikes between the regions was significantly correlated with oscillations in the high gamma range of the V1 LFP.

We also observed prominent contrast evoked beta/gamma oscillations in V1 below the narrowband high-gamma frequency which were not significantly synchronized with activity in dLGN. Recent reports show that V1 oscillations in this frequency range can be evoked by high contrast bars, and that the amplitude of these oscillations corresponds to the contiguous width of the bars (Veit et al., 2017a). Given that LFP is an aggregate measure of neuronal currents in a recording area (Buzsaki et al., 2012), this is consistent with the hypothesis that the low broadband gamma we observed in V1 reflects cortico-cortical interactions occurring somewhat independently of rhythmic thalamocortical influences. The evidence that these oscillations were mediated by local somatostatin-expressing interneurons further supports the hypothesis that low gamma oscillations reflect cortico-cortical neuronal communication in rodents (Veit et al., 2017a).

### Comparison to Studies in Other Species

Previous studies in awake rodents and primates have shown that LFP oscillations in separate frequency bands allow for single regions to selectively communicate with multiple others, as if by dedicated “channels” for transmission of sensory information (Colgin et al., 2009; Bastos et al., 2014). In the context of visual processing in primates, these distinct frequency bands are thought to correspond to mechanisms of top–down versus bottom–up signaling between lower- and higher-order visual cortical regions (Engel et al., 2001; Richter et al., 2017). Researchers have concluded that gamma-band oscillations in awake macaque V1 are an emergent property of cortico-cortical networks (Bastos et al., 2014) which serve to synchronize neuronal groups during information transfer between cortical areas (Bastos et al., 2015; Fries, 2015). This is supported by numerous primate studies in which cortical areas show inter-areal synchronization in the gamma band during visual processing (Bosman et al., 2012; Roberts et al., 2013; Lewis et al., 2016; Richter et al., 2017), while virtually no gamma-band activity is present in the thalamus (Bastos et al., 2014). Our results suggest that the rhythms of thalamocortical communication may be different in rodents, given that high gamma oscillations in the thalamo-recipient layer of mouse V1 are strongly associated with prominent rhythmic high gamma activity in dLGN. Previous studies which have explored the generation and function of gamma in the mouse retina, dLGN, and V1 further support this conclusion (Koepsell et al., 2009; Saleem et al., 2017; Storchi et al., 2017).

Similar investigations have been conducted in cats, although exclusively under anesthesia. In simultaneous recordings from the retina, LGN, and visual cortex, Castelo-Branco et al. (1998) identified rhythmic inter-areal spike correlations which fell largely into two gamma frequency bands. Higher frequency correlations in the 60–120 Hz range were reliably found within and between neurons of the LGN and retina, whereas cortico-cortical correlations were restricted to a lower 30–60 Hz frequency range. Interestingly, thalamocortical recording pairs exhibited correlations in both frequency ranges. In these cases, cortical neurons maintained a phase lag in high-frequency correlations, but a phase lead with correlations in the lower frequency. Altogether this suggests the presence of multiple synchronizing mechanisms in the cat visual system: most likely a feed-forward mechanism from structures early in the visual pathway, and a mechanism for parallel cortico-cortical or feedback cortico-thalamic synchronization (Castelo-Branco et al., 1998). Although direct comparison with our study may not be appropriate due to anesthesia, our findings are consistent with the concept of feed-forward synchronization outlined in this report.

Another important finding in this study was the shift in autocorrelation frequency of cortical neurons in response to stimulus motion (Castelo-Branco et al., 1998). These neurons were shown to fire rhythmically at 75 Hz in response to a static image, but slowed to 37 Hz when images were in motion. Simultaneous thalamic activity showed only a small positive shift in frequency. This may reflect a change in the balance of thalamic versus cortical input to the cortical neuron being observed as objects move across the visual field, resulting in sequences of cortical activity that are spatiotemporally relevant. This suggests that a moving stimulus may have changed the strength and/or frequency of the rhythmic correlations we observed, although the multi-band gamma responses we observe in layer 4 of V1 with a static checkerboard image are notably similar to those induced by a drifting grating in the study by Saleem et al. (2017). This establishes that cortical high gamma activity is present in mice in response to moving stimuli, as well as static high-contrast images and full-field luminance. Lastly, it is important to highlight that the cortical layer of cell recording was not noted in the study by Castelo-Branco et al. (1998), as cell depth undoubtedly influences the degree of connectivity with cortical versus thalamic neurons.

### Implications for the Processing of Visual Information

The emergence of distinct frequencies of gamma oscillations in the visual system remains an important focus of investigation into the neuronal mechanisms of visual processing. Since it is well-established that subthreshold and population-level oscillations modulate the effectiveness of communication between cortical neurons (Volgushev et al., 1998; Womelsdorf et al., 2007; Fries et al., 2007; Fries, 2015), it is intuitive that similar mechanisms would ensure that thalamocortical input is received with appropriate fidelity. But moreover, it is also very likely that critical information-processing operations occur during this relay. Some models of visual information coding predict that a common oscillation frequency between these regions, as well as spatial phase gradients within them, provides a framework for a neuronal population to encode and transmit information about stimulus properties via the phases of their action potentials (Nadasdy, 2009, 2010). In this interpretation, the common oscillation is at least partly dissociable from the action potential activity of the neurons carrying information, given that the underlying gradient of oscillatory activity is unperturbed by the encoded information. There is some physiological evidence to support this notion of functional specialization in the thalamocortical projection: First, inhibitory neurons of the thalamic reticular nucleus are activated by both thalamic and cortical neurons, and project back into the thalamic sensory nuclei, resulting in synchronized output from the thalamus that is timed according to activity in both regions (Jones, 2002; Sherman and Guillery, 2002). Secondly, in other mammals such as cats and monkeys, subpopulations of thalamic neurons are broadly-projecting and non-specific, with the hypothesized role of creating a synchronized matrix across the cortical sensory area (Jones, 2001). Third, sudden changes in the visual scene drive thalamic neurons to fire in bursts, thought to initiate a change in cortex that will make it receptive to subsequent input (Sherman and Guillery, 2002). Together, these factors may provide the physiological basis for synchronous oscillations to arise rapidly and create a spatiotemporal framework for phase coding. Our findings are consistent with this phase coding theory, although further investigation with greater spatial coverage is needed to determine whether cortical and thalamic high gamma oscillations occur with such a phase gradient.

## Conclusion

To the best of our knowledge, our results are the first derived from simultaneous recordings of LFP and spikes in V1 and dLGN in awake mice, which allowed us to directly link oscillations to spike synchrony in both areas. Our data show that stimulus properties have strong modulatory effects on the amplitude, bandwidth, and center frequency of these oscillations.

## Ethics Statement

This study was carried out in accordance with the recommendations of the University of Tennessee Health Science Center Institutional Animal Care and Use Committee (UTHSC IACUC). The protocol was reviewed and approved by the UTHSC IACUC.

## Author Contributions

SM designed and performed the experiments, developed and performed the data analysis, wrote the manuscript, and illustrated figures. YL contributed to experimental design and data analysis. MD provided critical assistance with data analysis. DH contributed to the experimental design, data interpretation, and the writing and critical revision of the manuscript.

## Conflict of Interest Statement

The authors declare that the research was conducted in the absence of any commercial or financial relationships that could be construed as a potential conflict of interest.
